# Model-guided design of mammalian genetic programs

**DOI:** 10.1126/sciadv.abe9375

**Published:** 2021-02-19

**Authors:** J. J. Muldoon, V. Kandula, M. Hong, P. S. Donahue, J. D. Boucher, N. Bagheri, J. N. Leonard

**Affiliations:** 1 Interdisciplinary Biological Sciences Program, Northwestern University, Evanston, IL 60208, USA.; 2 Department of Chemical and Biological Engineering, Northwestern University, Evanston, IL 60208, USA.; 3 Honors Program in Medical Education, Northwestern University Feinberg School of Medicine, Chicago, IL 60611, USA.; 4 Medical Scientist Training Program, Northwestern University Feinberg School of Medicine, Chicago, IL 60611, USA.; 5 Center for Synthetic Biology, Chemistry of Life Processes Institute, and Robert H. Lurie Comprehensive Cancer Center, Northwestern University, Evanston, IL 60208, USA.; 6 Departments of Biology and Chemical Engineering, University of Washington, Seattle, WA 98195, USA.

## Abstract

Genetically engineering cells to perform customizable functions is an emerging frontier with numerous technological and translational applications. However, it remains challenging to systematically engineer mammalian cells to execute complex functions. To address this need, we developed a method enabling accurate genetic program design using high-performing genetic parts and predictive computational models. We built multifunctional proteins integrating both transcriptional and posttranslational control, validated models for describing these mechanisms, implemented digital and analog processing, and effectively linked genetic circuits with sensors for multi-input evaluations. The functional modularity and compositional versatility of these parts enable one to satisfy a given design objective via multiple synonymous programs. Our approach empowers bioengineers to predictively design mammalian cellular functions that perform as expected even at high levels of biological complexity.

## Teaser

A suite of genetic components and computational models enables the predictive design of mammalian cellular functions.

## INTRODUCTION

Early demonstrations of genetically engineering customized functions in mammalian cells indicate a vast potential to benefit applications including directed stem cell differentiation (*1*, *2*) and cell-based therapies (*3*). In general, most applications require precise control of gene expression and the capability to sense and respond to external cues (*4*–*8*). Despite the growing availability of biological parts (such as libraries of promoters and regulatory proteins) that could be used to control cell states, assembling parts to compose customized genetic programs that function as intended remains a challenge, and it often requires iterative experimental tuning or down-selection to identify functional configurations. This highly empirical process limits the scope of programs that one can feasibly compose and fine-tune, as well as the performance of functional programs identified in this manner. Thus, the need for systematic and precise design processes represents a grand challenge in the field of mammalian synthetic biology.

Model-guided predictive design has been demonstrated in the composition of some cellular functions, including transcriptional logic in bacteria (*9*) as well as logical (*10*) and analog behaviors in yeast (*11*); however, this type of approach is less developed in mammalian systems. To date, transcription factors (TFs) based on zinc fingers (ZFs) (*12*, *13*), transcription activator-like effectors (TALEs) (*14*–*17*), dCas9 (*18*, *19*), and other proteins (*20*) have been used to implement transcriptional logic in mammalian cells. Some of these studies make use of protein splicing (*12*, *14*, *18*). Other studies have used RNA-binding proteins (*21*), proteases (*22*, *23*), and synthetic protein-binding domains (*17*). Yet, it remains difficult to predict circuit performance based only upon descriptions of the components. Associated challenges include the availability of appropriate parts (*24*), suitably descriptive models that support predictions using these parts (*25*), and computational and conceptual tools that facilitate the identification of designs that function robustly despite biological variability and cross-talk (*26*–*28*). In this study, we sought to address these issues by developing a model-driven process that enables one to propose a tractable set of candidate circuits for construction and testing without the need for empirical trial-and-error tuning. We validated this framework by using it to implement a variety of functions including digital and analog information processing, as well as sense-and-respond circuits.

## RESULTS

### Biological parts for integrating transcriptional and posttranslational control of gene expression

The strategy that we pursued for genetic program design was uniquely enabled by the COmposable Mammalian Elements of Transcription (COMET): a toolkit of TFs and promoters with tunable properties enabling precise and orthogonal control of gene expression (*13*). These TFs comprise a ZF DNA-binding domain and a functional domain, e.g., VP16 or VP64, which are activation domains (ADs) that with a ZF form an activator (ZFa). A protein including a ZF domain but lacking an AD functions as an inhibitor by competing with the cognate ZFa for binding. Promoters in this library contain ZF binding sites arranged in different configurations (e.g., ZF1x6-C has six compactly arranged ZF1 sites). Each combination of a promoter and a ZFa (and potentially an inhibitor) confers a characteristic level of transcriptional activity (fig. S1, A to E), and as part of this previous work, we developed mathematical models to characterize these relationships (*13*). Here, we investigate whether these biological parts and descriptive computational tools can be adapted and applied to achieve predictive genetic program design.

**Fig. 1:**
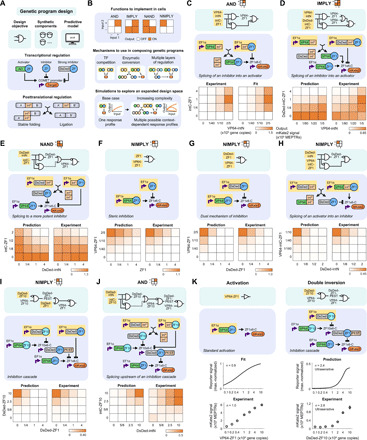
Logical evaluation is enabled by transcriptional and posttranslational regulation. (**A** and **B**) Cartoons depict (A) the genetic components and (B) their arrangement to produce intended functions. In the schematics, circles are protein domains, arrows indicate splicing or regulation, yellow highlighting denotes the inputs, and the red node is the output. (**C** to **J**) A panel of logic gates was designed, simulated, and experimentally evaluated. In the electronic diagrams (teal background), lines denote splicing or regulation. Processes that have a modest effect within the dose range examined, and that because of fundamentally analog behavior do not carry out a fully digital function, are denoted by dotted lines. In the mechanistic diagrams (blue background), purple bent arrows are promoters, and black arrows indicate splicing and regulation. Yellow highlighting denotes the components for which dose is varied (in gene copies). Simulation and experimental results are presented in heatmaps that indicate how the two inputs affect reporter output (mKate2 signal in MEPTRs). Color-coding denotes the mean reporter signal from three biological replicates (bar graphs in fig. S1N and histograms in fig. S1O), scaled by the maximum value in each heatmap. Simulations in (C) are from a fit to the data, and subsequent panels (D to J) are predictions. (**K**) Some of the motifs that were used in gate designs confer sharp transitions in output, e.g., two inhibitors in a cascade produced ultrasensitivity. The downstream inhibitor is tagged with a PEST degron.

Although COMET includes many parts for implementing transcriptional regulation, we hypothesized that complex genetic program design could be facilitated by introducing a mechanism for regulation at the posttranslational level (Fig. 1, A and B). To investigate this strategy, we evaluated new parts based on split inteins: complementary domains that fold and *trans*-splice to covalently ligate flanking domains (exteins) (*29*). We selected the split intein gp41-1 for its rapid splicing kinetics (*30*). To test an application of this mechanism, we appended an AD to the gp41-1 N-terminal fragment (intN) and a ZF to the C-terminal fragment (intC). These parts were used to construct an AND gate in which a reporter gene was induced only when both fragments were present (Fig. 1C and fig. S1, F and G), demonstrating that COMET-mediated gene expression can be adapted with splicing. We then incorporated this mechanism into our modeling framework by modifying ordinary differential equations from the original study (*13*), which concisely represent transcriptional regulation (Supplementary Text), and fitting newly introduced parameters to the data (fig. S1, H and I). We extended the model to describe parts in which split inteins were fused onto two types of inhibitors (fig. S1J): ZF, which competes with ZFa for binding site occupancy in the promoter, and ZF fused to DsRed-Express2 (abbreviated as DsRed-ZF), which also reduces the cooperativity of ZFa-mediated RNA polymerase II recruitment at multisite promoters (*13*). In addition, we introduced an R95K mutation to ablate the DsRed chromophore (*31*), yielding a nonfluorescent inhibitor we termed DsDed-ZF (fig. S1K). The extended model accurately recapitulated the component dose-dependent performance of the AND gate (Fig. 1C), providing verification that this extension can describe split intein–based circuits.

### Model-guided design of genetic programs

As a first test of the predictive capacity of the revised model, we simulated a panel of circuits that we hypothesized could carry out various logic operations (fig. S1L). Our objective was to identify promising designs for specific functions, so we opted not to include additional model complexity that might be required to predict all aspects of circuit behavior [e.g., potential cell burden effects (*32*)]. Throughout, simulations used a statistical model for gene expression variation, which we have previously shown to be important in accounting for the effect of cellular variation on how an engineered function is carried out across a cell population (Supplementary Text and fig. S1I) (*13*, *33*). From the panel, we selected several designs to test. First, to make an IMPLY gate, the AND gate was modified by appending DsDed to intC-ZF1 and coexpressing a VP64-ZF1 activator. Experimental outcomes (i.e., reporter readout across component doses) were consistent with the prediction that readout would be low only with DsDed-intC-ZF1 present in sufficient excess over its VP64-intN splicing partner to function as an inhibitor (Fig. 1D). To make a NAND gate, a DsDed-ZF1 inhibitor was split into DsDed-intN and intC-ZF1 and coexpressed with an activator. Outcomes were consistent with the prediction that readout would be low only with sufficient reconstitution of the inhibitor (Fig. 1E). These initial test cases demonstrate that model-guided design can identify effective topologies, as well as the precise relationship between input component levels and circuit output.

A versatile design framework would enable one to achieve a given performance objective via multiple circuits. We speculated that the combined properties of COMET and splicing-based extensions developed here might provide a sufficient basis for this capability. To investigate this possibility, we compared four designs for a NIMPLY gate, each of which uses a different mechanism (i.e., topology and/or choice of parts). The first two designs used inhibition mediated by ZF1 (Fig. 1F) or DsDed-ZF1 (Fig. 1G). The third design used splicing of a VP64-intC-ZF1 activator to a DsDed-ZF1 inhibitor such that the readout would be high only with VP64-intC-ZF1 in sufficient excess of its splicing partner DsDed-intN (Fig. 1H). The fourth design used a double inversion cascade, in which an upstream inhibitor prevented a downstream inhibitor from acting on the reporter (Fig. 1I); this scenario represents a variation on a topology that was previously examined in bacteria (*34*) and later in mammalian cells with dCas9-TFs (*35*). All four designs produced NIMPLY behavior as predicted. We next tested whether splicing could be combined with a cascade, and we were able to build an AND gate by splitting the cascade’s upstream inhibitor into DsDed-intN and intC-ZF10 (Fig. 1J). Unlike standard ZFa-mediated activation, this activation via double inversion exhibited ultrasensitivity (Hill coefficient *n* = 2.8)—a signal transformation in which a small change in input yields a large change in output, and high output is produced only with sufficient input (Fig. 1K and fig. S1M). Ultrasensitivity buffered the circuit against low inputs such that the output remained low for input levels that, in the standard activation case, would have produced half-maximal activation.

Across the panel, five of the eight gates exhibited a goodness of prediction metric (comparing all simulated and observed outcomes, *Q*^2^) of at least 0.90, where the maximum possible value is 1, indicating a high capacity for predicting dose response landscapes that had not been used in model training (fig. S1, P and Q). Even for the gate with the lowest *Q*^2^ (IMPLY; Fig. 1D and fig. S1P), the model correctly predicted the trend across most input dose combinations. Together, these results demonstrate the feasibility of model-guided design of logic gates in mammalian cells and that the choice of parts and mechanism yields predictable performance characteristics.

### Compression of circuit design using functional modularity

**Fig. 2:**
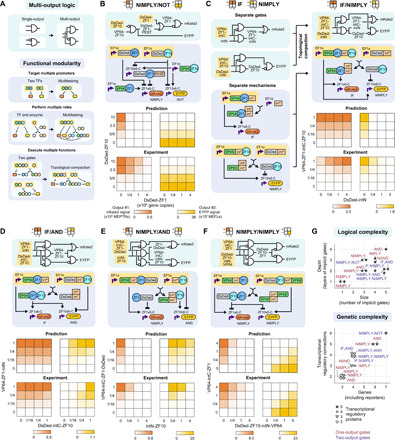
Compact multi-output logic is attained through functional modularity. (**A**) A strategy for multi-output logic is proposed by using multitasking proteins that retain the functions of their constituent domains. The cartoons depict the use of multiple DNA-binding domains on a TF to regulate multiple genes, the embedding of a split intein fragment within a functioning TF to enzymatically alter its activity, and the merging of features from multiple genetic programs to enable their compact simultaneous implementation. (**B** to **F**) A panel of MIMO gates was designed, simulated, and experimentally evaluated. As an example, (C) is deconstructed to show how separate topologies containing proteins that have some domains in common and are amenable to the appending of additional domains can be compressed. In the plots, color-coding denotes the mean mKate2 and enhanced yellow fluorescent protein (EYFP) reporter signal from three biological replicates (bar graphs in fig. S2F), scaled by the maximum value in each heatmap. (**G**) These plots summarize the complexity of the gates that were designed and validated in Figs. 1 (red) and 2 (purple), with complexity defined based on the size and depth of the circuits in the electronic diagrams (top) or based on the numbers of genes, regulatory connections, and regulatory proteins used (bottom). The expanded toolkit of genetic parts and model-guided approach were successful for building circuits spanning a range of attributes, which suggests that this design process could be executed reliably for many future objectives.

A putative advantage of orthogonal parts like COMET TFs and promoters is that these parts may be used together without disrupting their functions. However, simply appending modules can lead to inefficient and cumbersome designs, and thus, one focus of our approach was achieving genetic compactness as well as performance. Enhancing compactness could eliminate potential failure modes and reduce cargo size for gene delivery vehicles. Genetic compression—reducing the number of components for a given specification—has been investigated by using recombinase-mediated DNA rearrangement (*36*) and by borrowing from a software engineering strategy to eliminate redundancy (*37*). Here, we sought to implement a previously unexplored form of topological compaction based on protein multitasking (Fig. 2A). We hypothesized that because our genetic parts operate through direct interactions without relying on long-range mechanisms such as chromatin modification, they might exhibit functional modularity, i.e., domains could be concatenated and retain their functions. This property would be of great utility by enabling the use of multitasking proteins to act at multiple promoters or in both transcriptional and posttranslational roles to execute multiple functions in an efficient fashion.

We investigated whether functional modularity could enable the design of compact multi-input–multi-output (MIMO) systems. Ultimately, this capability could support the encoding of sophisticated decision-making strategies in which cells take different actions in different situations. As a base case, we simply appended a NIMPLY gate and a NOT gate in a noncompact manner, and the combination functioned as expected (Fig. 2B and fig. S2A). This success demonstrates the potential for composite functions, but it brings no efficiency relative to the individual gates. To test topological compaction, first, an IF/NIMPLY gate was proposed in which VP64-ZF1-intC-ZF10 would act as a bispecific activator (on two promoters) and interact with an inert DsDed-intN to produce a VP64-ZF1-intC/intN activator and a DsDed-ZF10 inhibitor (Fig. 2C and fig. S2B). The second gate, IF/AND, used an activator and an inhibitor to produce a bispecific activator and an inert protein, through essentially the inverse mechanism of that in the IF/NIMPLY gate (Fig. 2D and fig. S2C). Third, a NIMPLY/AND gate used a VP64-intC-ZF1-DsDed activator and an intN-ZF10 inhibitor to invert their respective activities (Fig. 2E and fig. S2D). We hypothesized that the former protein would act as an activator, in that DsDed would not preclude VP64 from conferring activation. Last, a NIMPLY/NIMPLY gate used two activators to produce a bifunctional inhibitor and an inert protein (Fig. 2F and fig. S2E). We note that if this circuit had used the same readout for both reporters, it would be a XOR gate. Overall, the model predictions explained most of the variance in experimental outcomes, and several cases were in close agreement (≥0.90 *Q*^2^) (fig. S2, F and G). Minor deviations are potentially attributable to effects such as differences in stability for different proteins; however, we chose not to incorporate such effects into the model because increasing model complexity could lead to overfitting. Moreover, the choice to simplify the description of protein stability did not preclude model-guided identification of high-performing designs.

Notably, when we examined performance at the single-cell level, some population-level outcomes were driven by subpopulations of cells. In some circuits, subpopulations induced one reporter or the other, but not both, and thus, population outcomes were driven by shifts in subpopulation frequencies (fig. S2, A, D, and E). In other circuits, this task distribution was not apparent (fig. S2, B and C). Although neither behavior was an explicitly designed feature, both types of behavior were predicted by simulations. Together, the gates described in Figs. 1 and 2 span a wide range of logical complexity (the number and the layers of implicit gates depicted in the electronic diagrams) and genetic complexity (the number of genes, regulatory connections, and regulatory proteins) (Fig. 2G). The successful development of these circuits without the need for additional tuning demonstrates that this framework may be well suited to overcoming complexity-associated barriers with mammalian genetic program design.

### Implementation of analog signal processing

Digital logic has uses in gates, switches, classifiers, and other decision-making functions (*38*), but we also wanted to examine whether our tools could be used to implement analog information processing. Fundamentally, the molecular interactions involved in implementing digital logic are also analog, because input and output concentrations span a continuous range, although the objective is for the output to be either high or low depending on each combination of high and/or low inputs. For analog circuits, we focused instead on properties that apply across the continuous input-output range. The first property that we sought to implement was ultrasensitivity, which is desirable in engineering sharp activation (*39*, *40*) and is observed in the natural control of processes including cell growth, division, and apoptosis (*41*). The second property was bandpass concentration filtering, in which an output is produced only when the input falls within a certain range (*22*, *42*). Bandpass concentration filtering is salient for both natural and synthetic spatial patterning (*43*) and has been used in directing cell differentiation (*1*).

To develop a strategy for implementing these properties, we made use of existing mechanistic insights. Previously, we determined that ZFa-mediated activation is cooperative at the level of transcription initiation, and in comparing promoter architectures, maximal transcription increased with the number and compactness of binding sites (*13*). This COMET promoter feature confers high inducibility as well as a high sensitivity to inhibition by proteins that compete for DNA binding. We also deduced that TF binding to promoters is generally noncooperative, and transcriptional output from such promoters is not inherently ultrasensitive to ZFa dose (Hill coefficient *n* = 1). To construct systems that do exhibit ultrasensitivity (*n* > 1), we examined several strategies in which the output is inhibited only at low activator doses (fig. S3, A to C). The first design made use of the inhibition conferred by intC-ZF1 before splicing with a VP16-intN input (fig. S3B). We reasoned that, at low VP16-intN doses, intC-ZF–mediated inhibition would dominate, and that at high doses, transactivation by reconstituted VP16-ZF would dominate. We also tested this concept with the addition of a DsDed-ZF to threshold the response by promoting relatively more inhibition at low input doses (fig. S3C). However, the increase in ultrasensitivity was modest for these cases, apparently from insufficient inhibition at low activator doses due to decreased protein stability caused by appending the intC domain to the inhibitory ZF (fig. S1H).

**Fig. 3:**
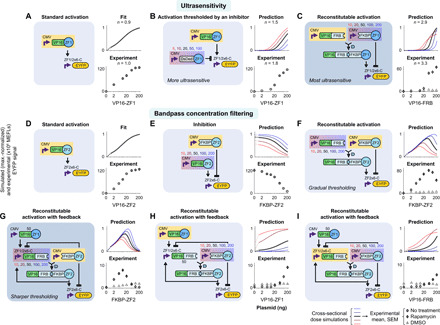
Analog behaviors are constructed by using TFs that play multiple roles. Reconstitutable TFs are conducive to (**A** to **C**) ultrasensitivity and (**D** to **I**) bandpass concentration filtering. Ultrasensitivity was increased by removing an inhibitor while producing an activator (C). For bandpass concentration filtering, a tight upper threshold was achieved with additional regulation in which moderate FKBP-ZF reconstitutes RaZFa and high FKBP-ZF inhibits the reporter and VP16-FRB (G). Simulations in (A) and (D) are fits to data, and other panels are predictions. Prediction plots indicate how output expression varies with the dose (nanograms of plasmid) of the component highlighted in yellow. Sets of simulated responses are for varying the component highlighted in red-to-blue gradation; each set contextualizes the “slice” that the tested response occupies in higher-dimensional component dose space. Each experiment plot corresponds to the simulation with the dark line. The ZF1/2x6-C promoter has six partially overlapping ZF1 and ZF2 sites. Dimethyl sulfoxide (DMSO) is the vehicle for rapamycin, which is used here as an environmental species (not an input). RaZFa simulations correspond to rapamycin treatment. Each experiment plot has either a no treatment series or both a rapamycin and a DMSO series. The mean and SEM of EYFP reporter signal are from three biological replicates (bar graphs in fig. S3, D to L). Axes are linearly scaled from 0 to 1 ng (dotted lines) and log-scaled above 1 ng. Linearly scaled versions of these plots are in fig. S3N.

Compared to a ZFa base case (*n* = 1.0) (Fig. 3A and fig. S3D), however, DsDed-ZF thresholding of ZFa-mediated activation increased *n* to 1.8 (Fig. 3B and fig. S3E). This outcome led us to consider a vehicular analogy: The circuits with DsDed-ZF are akin to applying the brake (inhibition) while applying the accelerator (activation), but a more effective approach might be to release the brake as the accelerator is applied. To realize this concept and circumvent choices that modulate protein stability, we used a chemically responsive COMET TF (RaZFa) in which rapamycin-induced heterodimerization domains FRB and FKBP are fused to an AD and a ZF, respectively. In the presence of rapamycin (which in this scenario is not an input, but rather an environmental species), heterodimerization of VP16-FRB and FKBP-ZF converts FKBP-ZF (brake) into RaZFa (accelerator), which induces the reporter. With rapamycin, the response of this circuit to VP16-FRB input exhibited greater ultrasensitivity (*n* = 3.3), consistent with the prediction (Fig. 3C and fig. S3F). Thus, in this system, ultrasensitivity can arise through cascades (Fig. 1) or reconstitution (Fig. 3), and neither mechanism requires the cooperativity in TF-DNA binding that is often associated with ultrasensitive responses.

We next investigated circuits to implement bandpass concentration filtering. Our strategy was to use mechanisms that inhibit reporter output only at high doses of activator input, and the predictions were based on a fitted ZFa base case (Fig. 3D and fig. S3G). We hypothesized that although FKBP-ZF is necessary for RaZFa-mediated activation, excess FKBP-ZF would be inhibitory. We confirmed that FKBP-ZF acted as an inhibitor (Fig. 3E and fig. S3H), and we implemented an RaZFa test circuit. The response to FKBP-ZF input showed a peak in output, but no sharp upper threshold, as predicted (Fig. 3F and fig. S3I). On the basis of these results, we designed a new topology to achieve a sharper bandpass. Of the regulation within this design, the two paths of negative regulation from FKBP-ZF (and not the positive feedback from RaZFa) appeared to be most important for sharpening the bandpass (fig. S3M). For the primary input to the bandpass, FKBP-ZF, we expected that at zero dose there would be no activation; at moderate doses there would be activation; and, at high doses excess FKBP-ZF would both decrease reconstitution (by inhibiting induction of VP16-FRB) and inhibit the reporter. The experimental outcomes closely matched the prediction of a bandpass with a sharp upper threshold (Fig. 3G and fig. S3J). Furthermore, when VP16-ZF or VP16-FRB doses were varied, the responses were activating as predicted (Fig. 3, H and I, and fig. S3, K and L), demonstrating a predictive capacity across multiple inputs for the system. These results demonstrate that our parts and approach are suitable for designing analog behaviors, as well as digital logic gates.

### Integration of genetic circuits with sensors to build sense-and-respond functions

While the predictive design of genetic programs is a substantial technical advance, using this capability to enable many potential applications will require integrating genetic circuits with native or synthetic parts that sense and modulate the state of the cell or its environment. A recurring challenge associated with this goal is level-matching the output of a sensor to the input requirements of a downstream circuit (*33*, *44*). We investigated whether our designed circuits could overcome this challenge and be effectively linked to sensors without requiring laborious trial-and-error tuning. Simulations suggested that adding an upstream layer of signal processing (i.e., for sensing) should be feasible because, in the model, ZFa can be arranged in series without prohibitively driving up background or dampening induced signal (fig. S4A).

**Fig. 4:**
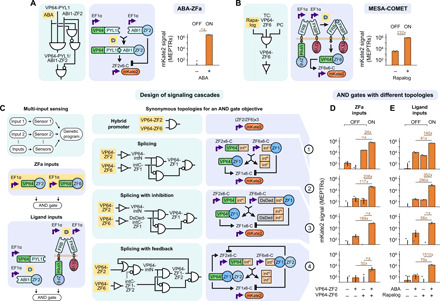
Sensors can be linked to genetic programs to make signaling cascades. MESA and COMET technologies can be combined to construct functional biosensors, and upstream biosensor output is well-matched to the requirements for downstream promoter input. (**A** and **B**) ABA-ZF2a and Rapa-MESA-ZF6a each exhibit ligand-inducible signaling (*P* = 2 × 10^−3^ and *P* = 1 × 10^−3^, respectively, one-tailed Welch’s unpaired *t* test). Ethanol is the vehicle for both ligands. For MESA, the TC contains an FRB ectodomain and intracellular COMET TF, and the PC contains an FKBP ectodomain and intracellular tobacco etch virus protease (TEVp). Each receptor chain contains a fibroblast growth factor receptor 4 (FGFR4) transmembrane domain. (**C** to **E**) Validated sensors were applied to implement multi-input sensing. AND logic was selected as a design goal, and four synonymous topologies—those that are intended to achieve the same goal through different mechanisms—were proposed and evaluated. For each input type (two columns for upstream ZFa or ligand sensing) and topology (four rows), reporter signal with two inputs differed from that with either or no input [*P* < 2 × 10^−16^ in each case, three-factor analysis of variance (ANOVA) and Tukey’s honest significant difference (HSD) test], indicating successful AND gate outcomes. Topologies 2 to 4 displayed negligible background signal (comparable to the signal with only the reporter present, ~10^1^ to 10^2^ MEPTRs; fig. S4H), despite involving multilayer signaling that can be a potential source of leak. The (ZF2/ZF6)x3 promoter has three pairs of alternating ZF2 and ZF6 sites. Bar graphs represent the mean, SEM, and values of mKate2 reporter signal from three biological replicates (depicted as dots; near-zero values are below the log-scaled *y*-axis lower limit). The numbers above bar pairs are the fold difference, and a fold difference of ∞ indicates that the denominator signal is less than or equal to zero.

We considered two classes of synthetic sensors (intracellular and transmembrane) for which we hypothesized that signaling (i.e., sensor output) could be coupled to COMET-based circuits. For the intracellular sensor, we built a new TF—ABA-ZFa, which is analogous to RaZFa—by fusing the abscisic acid (ABA)–binding domains PYL1 and ABI1 (*45*) to an AD and a ZF, respectively. For transmembrane sensing, we selected the modular extracellular sensor architecture (MESA)—a self-contained receptor and signal transduction system that transduces ligand binding into orthogonal regulation of target genes (*46*, *47*). In this mechanism, ligand-mediated dimerization of two transmembrane proteins called the target chain (TC) and protease chain (PC) promotes PC-mediated proteolytic *trans*-cleavage of a TC-bound TF. We explored several strategies for building COMET-compatible MESA based on a recently reported improved MESA design (*48*) and the parts developed in the current study (fig. S4, B to G). The best performance was observed using rapalog-inducible COMET-MESA that releases either ZFa for activating signaling or DsDed-ZF for inhibitory signaling (the latter represents a new function for MESA receptors) (fig. S4G); the ZFa-releasing COMET-MESA receptor was carried forward. We observed that both sensors displayed excellent performance in terms of reporter induction upon ligand treatment (Fig. 4, A and B). For ABA-ZF2a [ZF2a was selected for its potency stemming from cooperative transcriptional activation (*13*)], ligand-independent signal was unobservable, and induced signal was high, yielding perfect performance (Fig. 4A). For Rapa-MESA-ZF6a (ZF6a was also selected for its potency), the ligand-inducible fold difference in signal was ~200× (Fig. 4B), which is several-fold higher than was observed for recently reported receptors based on tTA (*48*) and also higher than the fold difference observed for a high-performing MESA that uses a distinct mechanism (*49*). Thus, Rapa-MESA-ZF6a is the highest performing MESA reported to date. Both sensors have a low off state and a high on state, apparently benefitting from the advantageous property of COMET promoter–based cooperativity.

We carried forward the two validated sensors and examined whether downstream circuits comprising genetic parts and designed topologies from this study could be seamlessly linked with the new input layer. To this end, we designed a panel of four synonymous topologies that implement AND logic through different mechanisms (Fig. 4C): (1) a hybrid promoter with alternating TF sites [based on a similar architecture from the original COMET study (*13*)], (2) splicing (as in Fig. 1C), (3) splicing with DsDed (as in Figs. 1D and 2D for tighter inhibition), and (4) and splicing with feedback (as in Fig. 3, G to I). All four topologies exhibited AND behavior when tested using ZFa as inputs (Fig. 4D), demonstrating the versatility for attaining a given objective in multiple ways. Moreover, when coupled to ligand-activated sensors, these circuits still conferred AND behavior, and performance was maintained (i.e., fold induction with two ligands remained much greater than with each ligand individually) in carrying out this more complex sensing function (Fig. 4E). A comparison across the designs provides some insights. The hybrid promoter in topology 1 was high-performing, and the splicing topologies in 2 to 4 generally yielded improvement over 1, despite the additional regulatory layer, by reducing the output generated from either single input alone to near reporter-only background (fig. S4H, shown with linear scaling). Of the topologies examined, 2 and 3 were the most effective at producing a high output when both inputs were present and low output when either input was present alone. These results demonstrate that genetic programs can be designed by a predictive model-driven process, and then these programs can be readily linked to different classes of sensors to implement high-performing sensing and processing functions.

## DISCUSSION

We developed an approach for accurate genetic program design by engineering new parts that combine transcriptional and posttranslational control and validating a computational modeling framework. The experimental observations closely matched simulations, even in scenarios using new proteins (including those with many domains) and new topologies (including those with many interacting parts), demonstrating a high predictive capacity across a range of complexity (Fig. 2G). Because the mechanisms used for binding, splicing, activation, and inhibition can be described by concise formalisms, no fundamental revamping (i.e., changing the underlying representation or granularity) of our original descriptive model was needed to enable predictions. Furthermore, no trial-and-error (e.g., empirical tuning of designs or substitution of parts) was needed to arrive at the specified design goals, which streamlined the design-build-test-learn cycle. We understand this to be possible because once the base case parts were characterized, no additional parameterization was needed to simulate how the parts would function when combined in new designs. Last, although a relatively small set of protein domains was used, we were able to combine the domains in many ways; a concise library was sufficient to produce the wide variety of behaviors observed.

This study benefited from insights that could facilitate future genetic circuit design efforts. Key strategies that enabled sophisticated design included the use of antagonistic bifunctionality (*50*), in which a component can exert opposing effects on a target gene depending on the other components in the circuit (fig. S4I), and functional modularity, which enabled multiple activities to be combined in individual proteins (fig. S4J). Sophisticated design was also enabled through the use of split genetic parts, including those that splice or dimerize. Split parts are conducive to encoding both digital (Figs. 1 and 2) and analog (Fig. 3) functions. Split parts also shift some of the regulation from the transcriptional level to the posttranslational level (i.e., protein-protein interactions), which could increase the speed of signal processing. Another benefit of split parts relates to circumventing cargo limitations of gene delivery vehicles, in that a large program that does not fit in one vector could be distributed across multiple vectors (*51*), in such a way that the parts interact to reconstitute the program only in cells receiving all of the vectors. We note that splicing and dimerization are complementary approaches with distinct consequences. In particular, splicing enables the swapping of protein domains, which does not constrain their subsequent localization to the same TF. This property was important for situations in which we removed a domain to add a function. For example, in Figs. 1H and 2 (C, E, and F), the AD was removed from the TF containing the DNA-binding domain to generate an inhibitor, and dimerization would not have provided this outcome. Last, we found that seamless level-matching could be achieved with multilayer circuits due to the potency of COMET TFs at cognate promoters, and in particular, the fusion of such a TF onto MESA resulted in the highest-performing version of this synthetic receptor to date (Fig. 4, B and E).

Together, these attributes and insights, in combination with the many ways in which components can be arranged to regulate each other, greatly expand the mammalian genetic program design space. In our current system, one can propose and formulate models for candidate designs based on principles for how the functionally modular parts operate. This process is model-guided in that circuits are proposed based on hypotheses and then vetted through simulations before construction and testing; in turn, through the process of model formulation and simulation, we gain intuition for making new hypotheses. In the future, it may be possible to further automate this process by conducting sweeps over large combinatorial spaces of topologies and identifying candidates that satisfy specified objectives. As fig. S1L illustrates, there are many ways that even a small number of protein domains can be arranged, and many distinct landscapes that can result, and so in future work there could be utility in conducting more systematic explorations. Such advances could further speed up the design process and broaden the scope of possible circuits and behaviors beyond those accessible solely by intuition. The new components and quantitative approaches developed here should enable bioengineers to build customized cellular functions for applications ranging from fundamental research to biotechnology and medicine.

## MATERIALS AND METHODS

### Split inteins

We expanded the COMET toolkit by incorporating gp41-1: a split intein that was identified putatively in a bioinformatic analysis (*52*), characterized in vitro and in *Escherichia coli* (*30*), and later used in mammalian cells (*53*). We maintained the TRSGY motif from the native sequence upstream of intN (at the end of the intN-adjoining extein), as done by (*30*, *54*–*56*) to retain high splicing activity; however, gp41-1 splicing has also been reported without this motif (*53*). We note that it is important to use cysteine as the first amino acid of intN (“1” site) and serine as the first amino acid downstream of intC (“+1” site) (*57*).

The protein sequence for intN was: CLDLKTQVQTPQGMKEISNIQVGDLVLSNTGYNEVLNVFPKSKKKSYKITLEDGKEIICSEEHLFPTQTGEMNISGGLKEGMCLYVKE, where the first amino acid is the “1” site, and TRSGY precedes this site.

The protein sequence for intC was: MMLKKILKIEELDERELIDIEVSGNHLFYANDILTHNS, where the last amino acid is the “+1” site.

The mutagenesis investigation (fig. S4) was informed by a crystal structure of the gp41-1 C1A catalytically inactive mutant (*58*). Electrostatic interactions between the β3 strand at the end of intN and the β6 strand at the start of intC were previously identified to form a charge zipper and proposed to be important in the capture and collapse mechanism that precedes splicing. In this mechanism, which was elucidated using the *Npu* DnaE split intein (*59*), capture involves electrostatic interactions between extended regions of the two fragments, and collapse involves compaction and stabilization of their initially disordered regions.

### Plasmid cloning and purification

Plasmids are listed in table S1. Plasmids were designed in SnapGene (GSL Biotech LLC), and primers were ordered from Integrated DNA Technologies. Several domains were sourced from Donahue *et al*. (*13*), but before the COMET study: VP16 and ZF domains are from Khalil *et al*. (*60*), VP64 is from Chavez *et al*. (*61*) (Addgene, #63798), FRB and FKBP are from Daringer *et al.* (*46*) (Addgene, #58876 and #58877), and DsRed-Express2 is a gift from D. Schaffer. Split inteins are from Hermann *et al*. (*53*) (Addgene, #51267 and #51268). The ABA-binding domains PYL1 and ABI1 (*62*–*66*) from Gao *et al*. (*45*) were used to make ABA-ZFa. The PEST sequence tag is from the mouse ornithine decarboxylase gene (*67*). Two types of plasmid backbones are used: pcDNA (pPD005; Addgene, #138749), which was modified from Thermo Fisher Scientific (#V87020) as described previously (*13*), and a series of transcription unit positioning vectors (TUPVs) derived from the modified pcDNA (*13*) and based upon the mMoClo system from Duportet *et al*. (*68*). Insulator sequences in TUPVs are from Bintu *et al*. (*69*) (Addgene, #78099).

Cloning was performed primarily using standard polymerase chain reaction (PCR), restriction, and ligation methods (reagents from New England BioLabs and Thermo Fisher Scientific) and, in some cases, through Golden Gate assembly, followed by transformation into chemically competent TOP10 *E. coli* (Thermo Fisher Scientific). Transformants were grown on LB/ampicillin agar plates at 37°C, colonies were picked and grown in liquid LB/ampicillin cultures, plasmid DNA was isolated (E.Z.N.A. plasmid mini kit, Omega Bio-tek), and DNA inserts were sequence-verified (ACGT Inc.). Plasmids were prepared using polyethylene glycol–based extraction as described previously (*13*). DNA purity and concentration were measured using NanoDrop 2000 (Thermo Fisher Scientific).

### Mammalian cell culture

Human embryonic kidney (HEK) 293FT cells were cultured in complete Dulbecco’s modified Eagle’s medium (DMEM) containing 1% DMEM powder (Gibco, #31600091), 0.35% (w/v) d-glucose (Sigma-Aldrich, #50-99-7), 0.37% (w/v) sodium bicarbonate (Thermo Fisher Scientific, #S233-500), 10% heat-inactivated fetal bovine serum (Gibco, #16140071), 4 mM l-glutamine (Gibco, #25030081), and penicillin (100 U ml^−1^) and streptomycin (100 μg ml^−1^) (Gibco, #15140122) in tissue culture–treated 10-cm dishes (Corning, #500001672) at 37°C in 5% CO_2_. To passage, medium was aspirated, and cells were washed in phosphate-buffered saline (PBS), incubated in trypsin-EDTA (Gibco, #25300054; 37°C, 5 min), detached by tapping the dish, and resuspended in fresh medium and plated. This cell line tested negative for *Mycoplasma* using the MycoAlert Mycoplasma Detection Kit (Lonza, #LT07-318).

### Transfection

Cells were plated in 24-well plates (Corning, #3524; 3 × 10^5^ cells ml^−1^, 0.5 ml per well) and transfected after adhering to the plates, typically between 8 and 14 hours after plating. Transfections were carried out using the calcium phosphate protocol (*13*): Plasmids are mixed together in defined amounts, CaCl_2_ (2 M; 15%, v/v) is added, and this solution is pipetted dropwise into an equal volume of 2× Hepes-buffered saline (500 mM Hepes, 280 mM NaCl, and 1.5 mM Na_2_HPO_4_); the solution is gently pipetted four times, and 3 min later, it is vigorously pipetted 20 times and added dropwise onto plated cells. In this study, DNA doses are reported in plasmid mass (ng) per well of cells or gene copies per well of cells. In each transfection experiment, “empty vector” (pPD005) was included in the transfection mix to maintain a consistent total mass of DNA per well. At 1 day after plating, medium was aspirated and replaced with fresh medium. In some experiments, the fresh medium contained vehicle or ligand. In Fig. 3, the vehicle was 0.1% dimethyl sulfoxide (DMSO) (v/v in cell culture) and the ligand was 100 nM rapamycin in 0.1% DMSO. In Fig. 4B, the vehicle was 0.1% ethanol (EtOH) and the ligand was either 100 nM rapalog (Takara, #AP21697) or 100 μM ABA (GoldBio, #21293-29-8) in 0.1% EtOH. In Fig. 4 (D and E), the vehicle was 0.2% EtOH, and the ligand conditions included 100 nM rapalog, 100 μM ABA, or both ligands in 0.2% EtOH. For fig. S4G, treatment was applied both before transfection and at the time of medium change to promote more immediate inhibitory signaling.

### Flow cytometry

Samples were prepared for flow cytometry generally at 40 to 48 hours after transfection. For each well, medium was aspirated, five drops of PBS were added, PBS was aspirated, and two drops of trypsin-EDTA were added. Cells were incubated (37°C, 5 min), plates were tapped to detach cells, and four drops of cold (4°C) DMEM were added. The contents of each well were pipetted up and down several times to detach cells and pipetted into fluorescence-activated cell sorting (FACS) tubes containing FACS buffer [2 ml; PBS (pH 7.4), 5 mM EDTA, and 0.1% (w/v) BSA]. Tubes were centrifuged (150*g*, 5 min), liquid was decanted, and two drops of FACS buffer were added. Samples were kept on ice and wrapped in foil, and then run on a BD LSR Fortessa special order research product using the following configuration: Pacific Blue channel with 405-nm excitation laser and 450/50-nm filter for EBFP2, fluorescein isothiocyanate (FITC) channel with 488-nm excitation laser and 505LP 530/30-nm filter for enhanced yellow fluorescent protein (EYFP), and phycoerythrin (PE)–Texas Red channel with 552-nm excitation laser and 600LP 610/20-nm filter for mKate2. Approximately 10^4^ live single-cell events were collected per sample.

### Flow cytometry data analysis

Flow cytometry data were analyzed using FlowJo software (FlowJo, LLC) to gate on single-cell (FSC-A versus FSC-H) and live (FSC-A versus SSC-A) bases, compensated using compensation control samples, and gated as transfection-positive (fig. S1A). The mean reporter signal in mean fluorescence intensity (MFI) was obtained for each sample. Ultra Rainbow Calibration Particles (Spherotech, #URCP-100-2H) were run in each flow cytometry experiment. Beads were gated on an FSC-A versus FSC-H basis, the nine bead subpopulations of varying intensities were identified, and the MFI for each subpopulation in the FITC channel and PE–Texas Red channel was obtained. These values in combination with manufacturer-supplied MEFL and MEPTR values for each subpopulation were used to fit a regression line with *y* intercept equal to zero. The mean and SEM for the three biological replicates were calculated. Autofluorescence background signal was subtracted using samples transfected with the transfection control marker, and error was propagated. MFI values were converted to MEFL or MEPTR using the slope of the regression line, and error was propagated. Histograms in supplementary figures represent reporter signal in MFI.

### Western blotting

HEK293FT cells were plated in six-well plates (6 × 10^5^ cells in 2 ml of DMEM per well). After the cells had adhered, cells were transfected using the calcium phosphate protocol with 400 μl of transfection mix per well. Each mix included 1.6 × 10^10^ copies per TF plasmid (pJM516, pJM554, or both; equivalent to 4 × 10^9^ copies per 0.5 ml in 24-well plates in functional assays), pJM451 transfection control, and pPD005 empty vector to maintain a consistent total mass of DNA per well. At 1.5 days after transfection, expression of EBFP2 transfection control was confirmed by fluorescence microscopy. Medium was aspirated, and cells were washed with cold PBS, lysed with radioimmunoprecipitation assay buffer [500 μl; 150 mM NaCl, 50 mM tris-HCl (pH 8), 1% Triton X-100, 0.5% sodium deoxycholate, and 0.1% SDS] containing protease inhibitor cocktail (Pierce/Thermo Fisher Scientific, #A32953), scraped from plate and pipetted into Eppendorf tubes, and incubated on ice for 30 min. Lysate was clarified by centrifugation (14,000*g*, 20 min, 4°C), and supernatant was collected in new tubes. A bicinchoninic acid assay (Pierce/Thermo Fisher Scientific, #23225) was performed in a 96-well plate (Thermo Fisher Scientific, #655906) using a microplate reader (BioTek Synergy H1), and protein concentrations were estimated based on a standard curve.

Each sample (5 μg of protein) was reduced in Laemmli buffer [final concentration, 60 mM tris-HCl (pH 6.8), 10% glycerol, 2% SDS, 100 mM dithiothreitol, and 0.01% bromophenol blue] at 70°C for 10 min, and then loaded onto 4 to 15% Mini-PROTEAN TGX precast gels (Bio-Rad, #4561086) and run (50 V for 10 min, then 100 V for 60 min). Wet transfer was performed onto Immuno-Blot PVDF membranes (Bio-Rad) at 100 V for 45 min. Protein transfer was confirmed by Ponceau S staining, and membranes were kept in tris-buffered saline (TBS; 50 mM tris, 138 mM NaCl, 2.7 mM KCl, and HCl to pH 8) until the blocking step. The following procedures for 3x-FLAG tag staining and hemagglutinin (HA) tag staining were conducted with the incubation steps on a rocker at room temperature. In the procedure for the 3x-FLAG tag, the membrane was blocked with 3% milk in TBS for 30 min, washed once with TBS for 5 min, incubated with primary antibody (mouse anti-FLAG M2, Sigma-Aldrich, #F1804; diluted 1:1000 in 3% milk in TBS) for 60 min, washed once with TBS for 5 min, washed twice with TBS containing 0.05% Tween [TBST (pH 8)] for 5 min each, incubated with secondary antibody [horseradish peroxidase (HRP)–anti-mouse immunoglobulin G (IgG), Cell Signaling Technology (CST), #7076; diluted 1:3000 in 5% milk in TBST (pH 7.6): 50 mM tris, 150 mM NaCl, HCl to pH 7.6, and 0.1% Tween] for 60 min, and washed three times with TBST (pH 7.6) for 5 min each. In the procedure for the HA tag, the membrane was blocked with 5% milk in TBST (pH 7.6) for 60 min, incubated with primary antibody [rabbit anti-HA, CST, #C29F4; diluted 1:1000 in 5% milk in TBST (pH 7.6)] for 60 min, washed three times with TBST (pH 7.6) for 5 min each, incubated with secondary antibody [HRP–anti-rabbit IgG, CST, #7074; diluted 1:3000 in 5% milk in TBST (pH 7.6)] for 60 min, and washed three times with TBST (pH 7.6) for 5 min each. The two membranes were incubated with Clarity Western ECL Substrate (Bio-Rad, #1705061) for 5 min and exposed to film, which was developed and scanned. Images were cropped, but no other image processing was performed.

### Nomenclature

Genetic programs for digital functions are depicted using genetic diagrams and electronic diagrams. The former represents each promoter, protein, and regulatory interaction, and the latter represents the logic underlying these interactions. Genes are named by their protein domains in order from N terminus to C terminus. Domains are generally connected by flexible linkers comprising glycine and serine. Several abbreviations are used: ZFa is an AD-ZF for any choice of AD and ZF; similarly, RaZFa is an AD-FRB and FKBP-ZF, and ABA-ZFa is an AD-PYL1 and ABI1-ZF. DsRed refers to wild-type DsRed-Express2, and DsDed is an DsRed-Express2 R95K mutant. We use a streamlined nomenclature that differs from that used in the original COMET report (*13*), in that inhibitors do not use ZFi notation: ZFi is now termed ZF, and DsRed-ZFi is now termed DsRed-ZF. The constitutive promoters used are cytomegalovirus (CMV) and EF1α. The inducible promoters used are COMET promoters, which are named as “[ZF domain]x[number of binding sites]-[binding site arrangement].” For example, ZF1x6-C has six compact sites for ZF1. There are two nonstandard cases: ZF1/2x6-C has six compact overlapping sites for ZF1 and ZF2 (up to six sites occupied, and up to six per ZF), and (ZF2/ZF6)x3 has six compact sites alternating between ZF2 and ZF6 (up to six sites occupied, and up to three per ZF). We note that some of the variation observed in the magnitude of readout signal across different circuits might be due to inherent properties of their topologies. One way to adjust the magnitude without having to examine alternative topologies would be to vary the number of TF binding sites in the promoter of the output gene, as investigated in the COMET study (*13*).

### Statistical analysis

Each sensor in Fig. 4 (A and B) was assessed using a one-tailed Welch’s unpaired *t* test, with the null hypothesis that reporter signal was equal with and without ligand treatment. Figure 4 (D and E) used a three-factor analysis of variance (ANOVA) and Tukey’s honest significant difference (HSD) test, with the null hypothesis that reporter signal was equal across the two input types, four topologies, and four input combinations. Effects were considered significant if *P* < 0.05, and additionally for the HSD test if the comparisons had an adjusted *P* < 0.05.

### Parameterization

Some parameter values are from the COMET study, and others are newly estimated or fitted (table S2).

### Ultrasensitivity

Ultrasensitivity is a type of nonlinear signal processing in which a small change in an input produces a large change in an output. We demonstrate how this property can be achieved with engineered motifs such as a double inhibition cascade (Fig. 1K), activation thresholded by an inhibitor (Fig. 3B), and reconstitutable activation (Fig. 3C). The ultrasensitivity of experimental and simulated dose responses is quantified using the Hill coefficient *n* from a modified Hill equation, in which *x* is the input dose (nanograms of plasmid or gene copies), *y* is the reporter signal, *y*_0_ is the reporter signal for zero input, and *a* and *b* are other fitted parameters. Standard ZFa dose responses are characterized by *n* ≈ 1. Ultrasensitive responses are those for which *n* > 1.

$$y=y_{0}+\frac{a\cdot x^{n}}{{\left(\frac{1}{b}\right)}^{n}+x^{n}}$$

### Goodness of fit

The *Q*^2^ metric was calculated analogously to the coefficient of determination (*R*^2^), but for a set of simulated and experimental outcomes. Maximal predictive capacity results in a *Q*^2^ value of 1, and lower predictive capacity results in lower values (*70*).

## SUPPLEMENTARY MATERIALS

Supplementary material for this article is available at http://advances.sciencemag.org/cgi/content/full/7/8/eabe9375/DC1

## Appendix

View/request a protocol for this paper from *Bio-protocol*.

## References

[1] P. Saxena, B. C. Heng, P. Bai, M. Folcher, H. Zulewski, M. Fussenegger, A programmable synthetic lineage-control network that differentiates human IPSCs into glucose-sensitive insulin-secreting beta-like cells. Nat. Commun. 7, 11247 (2016). 27063289 10.1038/ncomms11247 PMC4831023

[2] P. Guye, M. R. Ebrahimkhani, N. Kipniss, J. J. Velazquez, E. Schoenfeld, S. Kiani, L. G. Griffith, R. Weiss, Genetically engineering self-organization of human pluripotent stem cells into a liver bud-like tissue using Gata6. Nat. Commun. 7, 10243 (2016). 26732624 10.1038/ncomms10243 PMC4729822

[3] K. T. Roybal, L. J. Rupp, L. Morsut, W. J. Walker, K. A. McNally, J. S. Park, W. A. Lim, Precision tumor recognition by T cells with combinatorial antigen-sensing circuits. Cell 164, 770–779 (2016). 26830879 10.1016/j.cell.2016.01.011 PMC4752902

[4] M. A. Fischbach, J. A. Bluestone, W. A. Lim, Cell-based therapeutics: The next pillar of medicine. Sci. Trans. Med. 5, 179ps7 (2013). 10.1126/scitranslmed.3005568 PMC3772767 23552369

[5] T. Kitada, B. DiAndreth, B. Teague, R. Weiss, Programming gene and engineered-cell therapies with synthetic biology. Science 359, eaad1067 (2018). 29439214 10.1126/science.aad1067 PMC7643872

[6] M. Xie, M. Fussenegger, Designing cell function: Assembly of synthetic gene circuits for cell biology applications. Nat. Rev. Mol. Cell Biol. 19, 507–525 (2018). 29858606 10.1038/s41580-018-0024-z

[7] A. L. Slusarczyk, A. Lin, R. Weiss, Foundations for the design and implementation of synthetic genetic circuits. Nat. Rev. Genet. 13, 406–420 (2012). 22596318 10.1038/nrg3227

[8] F. Lienert, J. J. Lohmueller, A. Garg, P. A. Silver, Synthetic biology in mammalian cells: Next generation research tools and therapeutics. Nat. Rev. Mol. Cell Biol. 15, 95–107 (2014). 24434884 10.1038/nrm3738 PMC4032074

[9] A. A. K. Nielsen, B. S. Der, J. Shin, P. Vaidyanathan, V. Paralanov, E. A. Strychalski, D. Ross, D. Densmore, C. A. Voigt, Genetic circuit design automation. Science 352, aac7341 (2016). 27034378 10.1126/science.aac7341

[10] Y. Chen, S. Zhang, E. M. Young, T. S. Jones, D. Densmore, C. A. Voigt, Genetic circuit design automation for yeast. Nat. Microbiol. 5, 1349–1360 (2020). 32747797 10.1038/s41564-020-0757-2

[11] C. J. Bashor, N. Patel, S. Choubey, A. Beyzavi, J. Kondev, J. J. Collins, A. S. Khalil, Complex signal processing in synthetic gene circuits using cooperative regulatory assemblies. Science 364, 593–597 (2019). 31000590 10.1126/science.aau8287 PMC6650298

[12] J. J. Lohmueller, T. Z. Armel, P. A. Silver, A tunable zinc finger-based framework for Boolean logic computation in mammalian cells. Nucleic Acids Res. 40, 5180–5187 (2012). 22323524 10.1093/nar/gks142 PMC3367183

[13] P. S. Donahue, J. W. Draut, J. J. Muldoon, H. I. Edelstein, N. Bagheri, J. N. Leonard, The COMET toolkit for composing customizable genetic programs in mammalian cells. Nat. Commun. 11, 779 (2020). 32034124 10.1038/s41467-019-14147-5 PMC7005830

[14] F. Lienert, J. P. Torella, J.-H. Chen, M. Norsworthy, R. R. Richardson, P. A. Silver, Two- and three-input TALE-based AND logic computation in embryonic stem cells. Nucleic Acids Res. 41, 9967–9975 (2013). 23982518 10.1093/nar/gkt758 PMC3834826

[15] R. Gaber, T. Lebar, A. Majerle, B. Šter, A. Dobnikar, M. Benčina, R. Jerala, Designable DNA-binding domains enable construction of logic circuits in mammalian cells. Nat. Chem. Biol. 10, 203–208 (2014). 24413461 10.1038/nchembio.1433

[16] T. Lebar, A. Verbič, A. Ljubetič, R. Jerala, Polarized displacement by transcription activator-like effectors for regulatory circuits. Nat. Chem. Biol. 15, 80–87 (2019). 30455466 10.1038/s41589-018-0163-8

[17] Z. Chen, R. D. Kibler, A. Hunt, F. Busch, J. Pearl, M. Jia, Z. L. Van Aernum, B. I. M. Wicky, G. Dods, H. Liao, M. S. Wilken, C. Ciarlo, S. Green, H. El-Samad, J. Stamatoyannopoulos, V. H. Wysocki, M. C. Jewett, S. E. Boyken, D. Baker, De novo design of protein logic gates. Science 368, 78–84 (2020). 32241946 10.1126/science.aay2790 PMC7397813

[18] D. Ma, S. Peng, Z. Xie, Integration and exchange of split dCas9 domains for transcriptional controls in mammalian cells. Nat. Commun. 7, 13056 (2016). 27694915 10.1038/ncomms13056 PMC5063958

[19] H. Kim, D. Bojar, M. Fussenegger, A CRISPR/Cas9-based central processing unit to program complex logic computation in human cells. Proc. Natl. Acad. Sci. U.S.A. 116, 7214–7219 (2019). 30923122 10.1073/pnas.1821740116 PMC6462112

[20] B. Angelici, E. Mailand, B. Haefliger, Y. Benenson, Synthetic biology platform for sensing and integrating endogenous transcriptional inputs in mammalian cells. Cell Rep. 16, 2525–2537 (2016). 27545896 10.1016/j.celrep.2016.07.061 PMC5009115

[21] S. Ausländer, D. Ausländer, M. Müller, M. Wieland, M. Fussenegger, Programmable single-cell mammalian biocomputers. Nature 487, 123–127 (2012). 22722847 10.1038/nature11149

[22] X. J. Gao, L. S. Chong, M. S. Kim, M. B. Elowitz, Programmable protein circuits in living cells. Science 361, 1252–1258 (2018). 30237357 10.1126/science.aat5062 PMC7176481

[23] T. Fink, J. Lonzarić, A. Praznik, T. Plaper, E. Merljak, K. Leben, N. Jerala, T. Lebar, Ž. Strmšek, F. Lapenta, M. Benčina, R. Jerala, Design of fast proteolysis-based signaling and logic circuits in mammalian cells. Nat. Chem. Biol. 15, 115–122 (2019). 30531965 10.1038/s41589-018-0181-6 PMC7069760

[24] Z. Kis, H. S. Pereira, T. Homma, R. M. Pedrigi, R. Krams, Mammalian synthetic biology: Emerging medical applications. J. R. Soc. Interface 12, 20141000 (2015). 25808341 10.1098/rsif.2014.1000 PMC4424663

[25] N. Davidsohn, J. Beal, S. Kiani, A. Adler, F. Yaman, Y. Li, Z. Xie, R. Weiss, Accurate predictions of genetic circuit behavior from part characterization and modular composition. ACS Synth. Biol. 4, 673–681 (2014). 25369267 10.1021/sb500263b

[26] C. Briat, A. Gupta, M. Khammash, Antithetic integral feedback ensures robust perfect adaptation in noisy biomolecular networks. Cell Syst. 2, 15–26 (2016). 27136686 10.1016/j.cels.2016.01.004

[27] D. Del Vecchio, H. Abdallah, Y. Qian, J. J. Collins, A blueprint for a synthetic genetic feedback controller to reprogram cell fate. Cell Syst. 4, 109–120.e11 (2017). 28065574 10.1016/j.cels.2016.12.001 PMC5326680

[28] G. Lillacci, Y. Benenson, M. Khammash, Synthetic control systems for high performance gene expression in mammalian cells. Nucleic Acids Res. 46, 9855–9863 (2018). 30203050 10.1093/nar/gky795 PMC6182142

[29] M. Vila-Perelló, T. W. Muir, Biological applications of protein splicing. Cell 143, 191–200 (2010). 20946979 10.1016/j.cell.2010.09.031 PMC3004290

[30] P. Carvajal-Vallejos, R. Pallissé, H. D. Mootz, S. R. Schmidt, Unprecedented rates and efficiencies revealed for new natural split inteins from metagenomic sources. J. Biol. Chem. 287, 28686–28696 (2012). 22753413 10.1074/jbc.M112.372680 PMC3436554

[31] G. S. Baird, D. A. Zacharias, R. Y. Tsien, Biochemistry, mutagenesis, and oligomerization of DsRed, a red fluorescent protein from coral. Proc. Natl. Acad. Sci. U.S.A. 97, 11984–11989 (2000). 11050229 10.1073/pnas.97.22.11984 PMC17281

[32] T. Frei, F. Cella, F. Tedeschi, J. Gutiérrez, G.-B. Stan, M. Khammash, V. Siciliano, Characterization and mitigation of gene expression burden in mammalian cells. Nat. Commun. 11, 4641 (2020). 32934213 10.1038/s41467-020-18392-x PMC7492461

[33] R. M. Hartfield, K. A. Schwarz, J. J. Muldoon, N. Bagheri, J. N. Leonard, Multiplexing engineered receptors for multiparametric evaluation of environmental ligands. ACS Synth. Biol. 6, 2042–2055 (2017). 28771312 10.1021/acssynbio.6b00279 PMC5693605

[34] S. Hooshangi, S. Thiberge, R. Weiss, Ultrasensitivity and noise propagation in a synthetic transcriptional cascade. Proc. Natl. Acad. Sci. U.S.A. 102, 3581–3586 (2005). 15738412 10.1073/pnas.0408507102 PMC552778

[35] S. Kiani, J. Beal, M. R. Ebrahimkhani, J. Huh, R. N. Hall, Z. Xie, Y. Li, R. Weiss, CRISPR transcriptional repression devices and layered circuits in mammalian cells. Nat. Methods 11, 723–726 (2014). 24797424 10.1038/nmeth.2969 PMC4228775

[36] N. Lapique, Y. Benenson, Genetic programs can be compressed and autonomously decompressed in live cells. Nat. Nanotechnol. 13, 309–315 (2018). 29133926 10.1038/s41565-017-0004-z PMC5895506

[37] J. Beal, T. Lu, R. Weiss, Automatic compilation from high-level biologically-oriented programming language to genetic regulatory networks. PLOS ONE 6, e22490 (2011). 21850228 10.1371/journal.pone.0022490 PMC3151252

[38] Y. Benenson, Biomolecular computing systems: Principles, progress and potential. Nat. Rev. Genet. 13, 455–468 (2012). 22688678 10.1038/nrg3197

[39] E. C. O’Shaughnessy, S. Palani, J. J. Collins, C. A. Sarkar, Tunable signal processing in synthetic MAP kinase cascades. Cell 144, 119–131 (2011). 21215374 10.1016/j.cell.2010.12.014 PMC3035479

[40] T. Shopera, W. R. Henson, A. Ng, Y. J. Lee, K. Ng, T. S. Moon, Robust, tunable genetic memory from protein sequestration combined with positive feedback. Nucleic Acids Res. 43, 9086–9094 (2015). 26384562 10.1093/nar/gkv936 PMC4605329

[41] Q. Zhang, S. Bhattacharya, M. E. Andersen, Ultrasensitive response motifs: Basic amplifiers in molecular signalling networks. Open Biol. 3, 130031 (2013). 23615029 10.1098/rsob.130031 PMC3718334

[42] D. Greber, M. Fussenegger, An engineered mammalian band-pass network. Nucleic Acids Res. 38, e174 (2010). 20693530 10.1093/nar/gkq671 PMC2952875

[43] N. S. Scholes, M. Isalan, A three-step framework for programming pattern formation. Curr. Opin. Chem. Biol. 40, 1–7 (2017). 28463802 10.1016/j.cbpa.2017.04.008

[44] Y.-H. Wang, K. Y. Wei, C. D. Smolke, Synthetic biology: Advancing the design of diverse Genetic systems. Annu. Rev. Chem. Biomol. Eng. 4, 69–102 (2013). 23413816 10.1146/annurev-chembioeng-061312-103351 PMC3773533

[45] Y. Gao, X. Xiong, S. Wong, E. J. Charles, W. A. Lim, L. S. Qi, Complex transcriptional modulation with orthogonal and inducible dCas9 regulators. Nat. Methods 13, 1043–1049 (2016). 27776111 10.1038/nmeth.4042 PMC5436902

[46] N. M. Daringer, R. M. Dudek, K. A. Schwarz, J. N. Leonard, Modular extracellular sensor architecture for engineering mammalian cell-based devices. ACS Synth. Biol. 3, 892–902 (2014). 24611683 10.1021/sb400128g PMC4161666

[47] K. A. Schwarz, N. M. Daringer, T. B. Dolberg, J. N. Leonard, Rewiring human cellular input–output using modular extracellular sensors. Nat. Chem. Biol. 13, 202–209 (2017). 27941759 10.1038/nchembio.2253 PMC11536266

[48] H. I. Edelstein, P. S. Donahue, J. J. Muldoon, A. K. Kang, T. B. Dolberg, L. M. Battaglia, E. R. Allchin, M. Hong, J. N. Leonard, Elucidation and refinement of synthetic receptor mechanisms. Synth. Biol. 5, ysaa017 (2020). 10.1093/synbio/ysaa017 PMC7759213 33392392

[49] T. B. Dolberg, A. T. Meger, J. D. Boucher, W. K. Corcoran, E. E. Schauer, A. N. Prybutok, S. Raman, J. N. Leonard, Computation-guided optimization of split protein systems. bioRxiv 863530 [**Preprint**]. 5 December 2019. 10.1101/863530. PMC8084939 33526893

[50] Y. Hart, U. Alon, The utility of paradoxical components in biological circuits. Mol. Cell 49, 213–221 (2013). 23352242 10.1016/j.molcel.2013.01.004

[51] A. V. Anzalone, P. B. Randolph, J. R. Davis, A. A. Sousa, L. W. Koblan, J. M. Levy, P. J. Chen, C. Wilson, G. A. Newby, A. Raguram, D. R. Liu, Search-and-replace genome editing without double-strand breaks or donor DNA. Nature 576, 149–157 (2019). 31634902 10.1038/s41586-019-1711-4 PMC6907074

[52] B. Dassa, N. London, B. L. Stoddard, O. Schueler-Furman, S. Pietrokovski, Fractured genes: A novel genomic arrangement involving new split inteins and a new homing endonuclease family. Nucleic Acids Res. 37, 2560–2573 (2009). 19264795 10.1093/nar/gkp095 PMC2677866

[53] M. Hermann, P. Stillhard, H. Wildner, D. Seruggia, V. Kapp, H. Sánchez-Iranzo, N. Mercader, L. Montoliu, H. U. Zeilhofer, P. Pelczar, Binary recombinase systems for high-resolution conditional mutagenesis. Nucleic Acids Res. 42, 3894–3907 (2014). 24413561 10.1093/nar/gkt1361 PMC3973285

[54] D. Pan, B. Xuan, Y. Sun, S. Huang, M. Xie, Y. Bai, W. Xu, Z. Qian, An intein-mediated modulation of protein stability system and its application to study human cytomegalovirus essential gene function. Sci. Rep. 6, 26167 (2016). 27188239 10.1038/srep26167 PMC4870628

[55] J. A. Gramespacher, A. J. Stevens, D. P. Nguyen, J. W. Chin, T. W. Muir, Intein zymogens: Conditional assembly and splicing of split inteins via targeted proteolysis. J. Am. Chem. Soc. 139, 8074–8077 (2017). 28562027 10.1021/jacs.7b02618 PMC5533455

[56] J. K. Böcker, W. Dörner, H. D. Mootz, Light-control of the ultra-fast Gp41-1 split intein with preserved stability of a genetically encoded photo-caged amino acid in bacterial cells. Chem. Commun. 55, 1287–1290 (2019). 10.1039/c8cc09204d 30633261

[57] A.-L. Bachmann, H. D. Mootz, An unprecedented combination of serine and cysteine nucleophiles in a split intein with an atypical split site. J. Biol. Chem. 290, 28792–28804 (2015). 26453311 10.1074/jbc.M115.677237 PMC4661395

[58] H. M. Beyer, K. M. Mikula, M. Li, A. Wlodawer, H. Iwaï, The crystal structure of the naturally split gp41-1 intein guides the engineering of orthogonal split inteins from cis-splicing inteins. FEBS J. 287, 1886–1898 (2019). 31665813 10.1111/febs.15113 PMC7190452

[59] N. H. Shah, E. Eryilmaz, D. Cowburn, T. W. Muir, Naturally split inteins assemble through a “capture and collapse” mechanism. J. Am. Chem. Soc. 135, 18673–18681 (2013). 24236406 10.1021/ja4104364 PMC3865799

[60] A. S. Khalil, T. K. Lu, C. J. Bashor, C. L. Ramirez, N. C. Pyenson, J. K. Joung, J. J. Collins, A synthetic biology framework for programming eukaryotic transcription functions. Cell 150, 647–658 (2012). 22863014 10.1016/j.cell.2012.05.045 PMC3653585

[61] A. Chavez, J. Scheiman, S. Vora, B. W. Pruitt, M. Tuttle, E. P. R. Iyer, S. Lin, S. Kiani, C. D. Guzman, D. J. Wiegand, D. Ter-Ovanesyan, J. L. Braff, N. Davidsohn, B. E. Housden, N. Perrimon, R. Weiss, J. Aach, J. J. Collins, G. M. Church, Highly efficient Cas9-mediated transcriptional programming. Nat. Methods 12, 326–328 (2015). 25730490 10.1038/nmeth.3312 PMC4393883

[62] S.-Y. Park, P. Fung, N. Nishimura, D. R. Jensen, H. Fujii, Y. Zhao, S. Lumba, J. Santiago, A. Rodrigues, T.-F. F. Chow, S. E. Alfred, D. Bonetta, R. Finkelstein, N. J. Provart, D. Desveaux, P. L. Rodriguez, P. M. Court, J.-K. Zhu, J. I. Schroeder, B. F. Volkman, S. R. Cutler, Abscisic acid inhibits type 2C protein phosphatases via the PYR/PYL family of START proteins. Science 324, 1068–1071 (2009). 19407142 10.1126/science.1173041 PMC2827199

[63] Y. Ma, I. Szostkiewicz, A. Korte, D. Moes, Y. Yang, A. Christmann, E. Grill, Regulators of PP2C phosphatase activity function as abscisic acid sensors. Science 324, 1064–1068 (2009). 19407143 10.1126/science.1172408

[64] K. Melcher, L.-M. Ng, X. E. Zhou, F.-F. Soon, Y. Xu, K. M. Suino-Powell, S.-Y. Park, J. J. Weiner, H. Fujii, V. Chinnusamy, A. Kovach, J. Li, Y. Wang, J. Li, F. C. Peterson, D. R. Jensen, E.-L. Yong, B. F. Volkman, S. R. Cutler, J.-K. Zhu, H. E. Xu, A gate–latch–lock mechanism for hormone signalling by abscisic acid receptors. Nature 463, 602–608 (2009). 10.1038/nature08613 PMC2810868 19898420

[65] K.-i. Miyazono, T. Miyakawa, Y. Sawano, K. Kubota, H.-J. Kang, A. Asano, Y. Miyauchi, M. Takahashi, Y. Zhi, Y. Fujita, T. Yoshida, K.-S. Kodaira, K. Yamaguchi-Shinozaki, M. Tanokura, Structural basis of abscisic acid signalling. Nature 462, 609–614 (2009). 19855379 10.1038/nature08583

[66] P. Yin, H. Fan, Q. Hao, X. Yuan, D. Wu, Y. Pang, C. Yan, W. Li, J. Wang, N. Yan, Structural insights into the mechanism of abscisic acid signaling by PYL proteins. Nat. Struct. Mol. Biol. 16, 1230–1236 (2009). 19893533 10.1038/nsmb.1730

[67] P. Loetscher, G. Pratt, M. Rechsteiner, The C terminus of mouse ornithine decarboxylase confers rapid degradation on dihydrofolate reductase. J. Biol. Chem. 17, 11213–11220 (1991). 2040628

[68] X. Duportet, L. Wroblewska, P. Guye, Y. Li, J. Eyquem, J. Rieders, T. Rimchala, G. Batt, R. Weiss, A platform for rapid prototyping of synthetic gene networks in mammalian cells. Nucleic Acids Res. 42, 13440–13451 (2014). 25378321 10.1093/nar/gku1082 PMC4245948

[69] L. Bintu, J. Yong, Y. E. Antebi, K. M. Cue, Y. Kazuki, N. Uno, M. Oshimura, M. B. Elowitz, Dynamics of epigenetic regulation at the single-cell level. Science 351, 720–724 (2016). 26912859 10.1126/science.aab2956 PMC5108652

[70] V. Consonni, D. Ballabio, R. Todeschini, Evaluation of model predictive ability by external validation techniques. J. Chemometr. 24, 194–201 (2010).

